# Patterns of somatic distress among conflict-affected persons in the Republic of Georgia

**DOI:** 10.1016/j.jpsychores.2015.01.015

**Published:** 2015-05

**Authors:** Ruben Moreno Comellas, Nino Makhashvili, Ivdity Chikovani, Vikram Patel, Martin McKee, Jonathan Bisson, Bayard Roberts

**Affiliations:** a Faculty of Public Health and Policy, London School of Hygiene and Tropical Medicine, London, United Kingdom; b Global Initiative on Psychiatry–Tbilisi, Tbilisi, Georgia; c Ilia State University, Tbilisi, Georgia; d Curatio International Foundations, Tbilisi, Georgia; e Cardiff University School of Medicine and Cardiff and Vale University Health Board, Cardiff, United Kingdom; f Faculty of Epidemiology and Population Health, London School of Hygiene and Tropical Medicine, London, United Kingdom

**Keywords:** Georgia, Mental, Somatic distress, War, Armed conflict, Forced displacement, Internally displaced

## Abstract

**Background:**

There are substantial risk factors for somatic distress (SD) among civilian populations affected by armed conflict in low and middle income countries. However, the evidence is very limited. Our aim was to examine patterns of SD among conflict-affected persons in the Republic of Georgia, which has over 200,000 internally displaced persons (IDPs) from the wars over separatists regions in the 1990s and with Russia in 2008.

**Methods:**

A cross-sectional household survey was conducted with 3600 randomly selected IDPs and former IDPs (returnees). SD was measured using the Patient Health Questionnaire (PHQ-15). Post-traumatic stress disorder (PTSD), depression, anxiety, and disability were measured using the Trauma Screening Questionnaire, Patient Health Questionnaire 9, Generalised Anxiety Disorder 7, and WHO Disability Assessment Schedule 2.0, respectively. Descriptive, tetrachoric and multivariate regression analyses were used.

**Results:**

Forty-two percent of respondents (29% men; 48% women) were recorded as at risk of SD (PHQ-15 score > 5). In tetrachoric analysis, SD scores were highly correlated with depression (*r* = 0.60; *p* < 0.001), PTSD (*r* = 0.54; *p* < 0.001), and anxiety (*r* = 0.49; *p* < 0.001). Factors significantly associated with SD in the multivariate regression analysis were depression, PTSD, anxiety, individual trauma event exposure, cumulative trauma exposure, female gender, older age, bad household economic status, and being a returnee compared to an IDP. SD was also associated with increased levels of functional disability (*b* = 6.73; *p* < 0.001).

**Conclusions:**

The high levels of SD among IDPs and returnees in Georgia indicate significant suffering. The findings have implications for both mental and physical health services in Georgia.

## Highlights

• Exposure to armed conflict poses substantial risk for somatic distress.
• Very little evidence on somatic distress among conflict-affected civilians.
• Nearly half of women and a third of men in our study were recorded as at risk of somatic distress.
• Somatic distress was associated with trauma exposure, mental disorders, and disability.
• Such high levels of somatic distress have implications for health services in Georgia.

## Background

Almost 50 million people have been forcibly displaced from their home areas by armed conflict globally, the vast majority of whom live in low- and middle-income countries. These comprise over 33 million internally displaced persons (IDPs) who remain within the borders of their countries and over 16 million refugees and stateless persons who are living in other countries [26,47]. There are millions more civilians who are resident in conflict-affected areas or in places that were, until recently, beset by conflict. High rates of mental disorders such a post-traumatic stress disorder (PTSD), depression and anxiety have been reported among conflict-affected civilian populations due to exposure to violent and traumatic events, impoverishment, poor living conditions, and other daily stressors [32,39,46]. However, less attention appears to have been paid to somatic distress (SD).

SD is characterized by symptoms that suggest physical illness or injury but which cannot be explained fully by a general medical condition or by the direct effect of a substance, or by another mental disorder [1]. The types and meaning of somatic symptoms vary between cultures, with each culture having particular beliefs on the meaning of somatic symptoms [19]. SD commonly gives rise to a high burden on individuals as well as health services [2,5,17,41]. While research on SD is complicated by variation in the definition and measurement of SD [6,11,14] and poorly understood pathophysiological mechanisms ([31], Clauw, Engel, et al. 2003, [11]), SD has been shown to be a valid construct from a transcultural perspective [17].

SD might be expected to be common in conflict-afflicted populations given the high levels of known risk factors for its development such as exposure to traumatic events, existing mental disorders, and socioeconomic deprivation [10,22,38,46]. The importance of studying SD in traumatized populations has been highlighted, particularly from a transcultural perspective [22]. Such work highlights how SD can be generated by trauma associations, arousal, and catastrophic cognitions [21,22]. Yet despite the high frequency of potential risk factors for SD, there is very limited evidence on SD among conflict-affected civilian populations in low- and middle-income countries, reflecting the limited evidence base on SD in low- and middle-income settings more generally [38,43,50], with most of the existing evidence limited to high-income countries [18,20,37]. Two relevant exceptions include a study on levels of SD among 1574 primary care users in post-conflict Bosnia [5], and a cross-sectional study of 163 Kosovar civilian war survivors that analyzed the relationship between SD, exposure to traumatic events, and disability [35].

In this study, we seek to narrow this gap by using data we collected for a study on mental disorders among conflict-affected persons in the Republic of Georgia. Georgia has been afflicted by armed conflict multiple times in the last few decades. The first phase of conflict began in the early 1990s following separatist movements in Abkhazia and South Ossetia, leading to over 300,000 people being internally displaced, of whom around 200,000 have not yet returned to their homes. The second main phase arose from the conflict with Russia over South Ossetia in August 2008 in which over 120,000 ethnic Georgians were displaced elsewhere in Georgia and around 20,000 remain as IDPs. Despite the high numbers of conflict-affected persons in Georgia, there have been very few epidemiological studies there on mental health and none on SD. The risk factors for SD were potentially present among conflict-affected persons in Georgia, such as exposure to traumatic events, elevated mental disorders, and socioeconomic deprivation. In addition, anecdotal reports from conflict-affected persons and health workers in Georgia had indicated the presence of unexplained symptoms. Understanding levels of SD among the conflict-affected population in Georgia could help identify previously undocumented suffering and inform responses.

The overall aim of the study was to examine patterns of SD among conflict-affected persons in the Republic of Georgia. The specific objectives were (i) to measure levels of SD; (ii) to examine association of mental disorders, trauma exposure, and demographic and socioeconomic characteristics with SD; and (iii) to examine the association between SD and functional disability.

## Methods

### Data collection

The project used a cross-sectional survey design and multi-stage random sampling with stratification by region and displacement status, seeking maximum representation of the conflict-affected populations in Georgia. A total sample size of 3,600 was determined to provide adequate statistical power for the overall study and consisted of 1,200 respondents from each of the three main conflict-affected populations groups in Georgia: those displaced from the conflicts in the 1990s (“1990s IDPs”), those displaced from the 2008 conflict (“2008 IDPs”), and former IDPs from the 2008 conflict who have returned to their home areas (“returnees”).

Three hundred and sixty primary sampling units (PSUs) (120 PSUs for each of the three study population groups of 1990 IDPs, 2008 IDPs, and returnees) were selected based on probability proportion to size, using a sampling frame from data provided by the Ministry of Internally Displaced Persons and the Governor's office of the Shida Kartli region. The number of PSUs was selected to meet the statistical requirements of the overall study, particularly for conducting multilevel modeling used in a separate analyses [42]. The random walk method was then used to randomly select households in each primary sampling unit. This involved selecting a random starting direction from a central location in the cluster, with households lying on this transect from the center to the border of the cluster counted, with one of them then chosen at random and the next X nearest households subsequently visited [53]. Within the selected household, a member of the household (aged ≥ 18 years) was randomly selected for interview based on nearest birthday. Up to three visits were made on different days and times if the household was empty or selected respondent not available. After the third attempt, a replacement household was visited. Trained fieldworkers conducted face-to-face interviews in the respondents homes, with all interviews held in Georgian. Data collection took place between October and December of 2011. The response rate was 79%. Informed consent was provided by all respondents. Ethics approval was provided by the National Council on Bioethics in Georgia and the Ethics Committee of the London School of Hygiene and Tropical Medicine.

### Measurement

Somatic symptoms were measured with the widely used 15-item Patient Health Questionnaire (PHQ-15) [28,51]. To test the psychometric properties of the PHQ-15 with the study sample, we conducted a factor analysis that revealed a solid structure of 1 factor (eigenvalue = 4.6). All items had relatively high loadings to factor one (0.42 to 0.72), except for items 4 (menstruation problems) and 5 (pain during intercourse), which had factor loadings of 0.13 and 0.14, respectively. This is consistent with previous validation studies recommending exclusion of both items from analysis [51], which we did. Following the PHQ-15 guidelines, symptoms were scored as 0 (“not bothered at all”), 1 (“bothered a little”), or 2 (“bothered a lot”), except for fatigue and sleep disturbance, which were scored as 0 (“not at all”), 1 (“several days”), or 2 (“more than half the days” or “nearly every day”). A final score is calculated by summing each item, and based on the PHQ-15 guidelines, a total score ≥ 15 indicates high SD severity, while a score of > 5 indicates risk of SD and this is the recommended and most commonly cutoff for the PHQ-15 [28,51].

PTSD was measured using the Trauma Screening Questionnaire (TSQ), which consists of 10 items on PTSD symptoms over the past 1 week, with *No* (= 0) and *Yes* (= 1) responses, which are summed to produce an overall score range of 0–10, with the TSQ's cutoff of > 5 used to indicate possible PTSD [4,52]. Depression was measured using the Patient Health Questionnaire (PHQ-9), which consists of 9 questions on depression symptoms over the last 2 weeks, with responses of *not at all* (= 0), *several days* (= 1), *more than half the days* (= 2), and *nearly every day* (= 3), with item scores summed to produce a total score range of 0–27, with the PHQ-9's suggested cutoff of ≥ 10 used to indicate at least moderate depression [27]. Anxiety was measured using the Generalised Anxiety Disorder (GAD-7) instrument, which consists of 7 questions on anxiety symptoms over the last 2 weeks, with the same response options and scoring as the PHQ-9, producing a total score range of 0–21, with the GAD-7's suggested cutoff of ≥ 10 used to indicate at least moderate anxiety [45]. Functional disability was assessed using the WHO Disability Assessment Schedule (WHODAS 2.0) (12 items version), which consists of 12 items on six activity domains for functional disability (cognition, mobility, self-care, getting along, life activities, and participation) with a recall period of the previous 30 days, with response option scores ranging from 0 (*none*) to 4 (*severe*). These are recoded to produce a general disability score which is rescaled from 0–36 to 0–100 (with higher scores representing higher levels of disability) [48,49].

The study instruments were translated using standard procedures involving the following: (i) translation from English into Georgian using professional translators, with translations reviewed by Georgian mental health experts individually and then as a group for cultural relevance, content and concept consistency, clarity, and understanding; (ii) a back-translation to check for accuracy, consistency, and equivalence, with adjustments made accordingly; and (iii) piloting and field testing to refine the instruments further.

The PHQ-15 showed good validity and reliability. The factor analysis described above indicates good construct validity. For known groups validity (convergent validity), higher levels of exposure to traumatic events and mental disorders correlated with higher levels in the PHQ-15 score (see below), as commonly seen elsewhere [35,44].Similarly, the PHQ-15 SD score was closely correlated with the scores of the instruments for PTSD, depression, and anxiety (see below). For reliability, we conducted a separate mini survey of 110 randomly selected IDPs living in Tbilisi (not included in the main survey) in which the PHQ-15 and other instruments were administered to the same respondent 4 days apart in order to assess the test-retest reliability of the instruments. The intraclass correlation of the PHQ-15 for the test–retest was extremely strong at 0.97. There was also strong internal reliability, with a Cronbach's alpha score of 0.83.The other instruments were also found to be valid and reliable, with the details reported elsewhere [30].

Lifetime exposure to violent and traumatic events was measured using an adapted version of the Harvard Trauma Questionnaire (HTQ) [33,34] (see Table 2 for selected items). Items from the HTQ were treated as both individual items and cumulatively (0, 1, 2, ≥ 3). The questionnaire also included a range of demographic and socioeconomic items such as gender, age, education level, displacement status and history, general living conditions, and conditions in the community, household deprivation, and current household economic situation.

### Data analysis

To estimate the strength of association between SD, exposure to traumatic events, mental disorders, and socioeconomic characteristics, multivariate regression analysis was used, with a binary outcome of “being at risk of SD” (PHQ-15 score > 6). Given the known risk factors for SD from other settings, the model adjusted for variables for mental disorders, gender, age, educational attainment, marital status, employment status, and household economic status (with respondents rating their household economic status as very good, good, average, bad, very bad which were then combined to very good/good, average, bad/very bad for the analysis) [5,35,38], with displacement status also added for the purposes of this study. We also examined comorbidity between SD and other mental disorders using tetrachoric correlations.

To examine the influence of SD on disability, a linear multivariate regression model was run using disability (WHODAS-2) as a continuous scale, with the main exposure variable that being “at risk” of SD (i.e., PHQ-15 score ≥ 6). The model was adjusted for displacement status, sex, age, economic status, and the mental disorders of PTSD, depression, and anxiety.

Statistical significance was assumed at *p* < 0.05. Data were weighted to reflect the actual proportions of “1990s IDPs”, “2008 IDPs,” and “returnees” in the overall conflict-affected population of Georgia. Data were also adjusted for the cluster survey design. Analysis was carried out using STATA 12.0.

## Results

Our sample consisted of 3600 respondents, 34% of whom were male and 65% female, reflecting findings of studies of the general population in Georgia as many men have left to find employment elsewhere [7]. The respondent characteristics are given in Table 1. The average age was 48.4, with an even distribution between age categories (30–35%) in the 18–39, 50–64, ≥ 65 categories. Most respondents had reached secondary education (69%), lived in government owned accommodation (57%), and had average (36%) to bad (52%) economic status.

Nearly a quarter of respondents (23%) were categorized as having PTSD (19% men; 25% women), 13% with depression (10% men; 14% women), and 11% for anxiety (8% men; 11% women) (Table 1). Further details on the distribution of mental disorders and their comorbidity are reported elsewhere [30].

The mean SD score was 5.39 [95% CI 5.24; 5.53] (men 4.0 [95% CI 3.78; 4.22]; women 5.87 [95% CI 5.69; 6.05]). A total of 41.7% of the study respondents were recorded as being at risk (PHQ-15 > 5) of SD (29% men and 48% women) (Table 2). The distribution of individual SD symptoms is given in Online Appendix 1.

More than 40% experienced first-hand one or several combat situations as well as lack of shelter (45%) (Table 3). Similarly, 25% experienced the death of a family member or close friend during conflict, while almost 20% of the sample witnessed murder, violence, and acts against family and friends. Almost 17% experienced serious injury. A quarter of respondents experienced one type of trauma exposure, 21% experienced two types, and almost 34% experienced three or more PTEs, while around 20% did not experience any of the types of trauma exposure included in the survey. Table 3 also shows the prevalence of the trauma exposure event in those with and without SD.

The adjusted associations between trauma exposure and SD are shown in the multivariate model in Table 4. Of the individual events, serious injury, being in a combat situation, witnessing murder/torture of strangers, and experiencing death of family member were all positively associated with SD. The cumulative trauma exposure event data indicate a dose–response relationship, with greater cumulative trauma exposure showing a stronger association with SD.

In terms of the relationship between SD and mental disorders, 8.8% of respondents were at risk of PTSD-SD comorbidity, 6.7% depression-SD comorbidity, and 4.7% anxiety-SD comorbidity. The tetrachronic correlations among dichotomized scores (high risk = 1, low risk = 0) were all positive and statistically significant at the *p* < 0.001, suggesting an important degree of comorbidity. SD scores highly correlated with depression (*r* = 0.60; *p* < 0.001), PTSD (*r* = 0.54; *p* < 0.001), and anxiety (*r* = 0.49; *p* < 0.001). When adjusted for the influence of demographic, socioeconomic, and trauma exposure factors in the multivariate analysis, they were still significantly associated with SD (PTSD OR < 2.66; depression OR < 3.87; anxiety < 1.76) (Table 4).

Other factors significantly associated with SD (Table 4) included gender, with women over twice as likely to be at risk of SD (OR < 2.51). Similarly, older age increased the risk of SD. However, lower education level appeared protective against SD, as did being single (OR < 0.68). However, bad household economic status was associated with higher risk of SD (OR < 2.44). Displacement status was associated with SD risk, with returnees at higher risk of SD compared to 2008 IDPs and 1990s IDPs (but there was no significant difference between 2008 IDPs and 1990s IDPs).

We also examined the association of trauma exposures, mental health, and sociodemographic variables with medium (PHQ-15 Score > 9) and high (PHQ-15 Score > 15) SD, and these are shown in the Online Appendix 2. These indicate higher risk among those who experienced and individual cumulative traumatic events; those with mental disorders, women; older age; and returnees.

SD risk was significantly associated with increased levels of functional disability (*b* = 6.73 [95% CI 5.25; 8.20] *p* < 0.001), after adjusting for gender, age, education, economic status, IDP status, trauma exposure, and mental disorders in a multivariate analysis. High SD (PHQ-15 > 15) remained significantly associated with functional disability (*b* = 5.50 [95% CI 0.66; 10.33], *p* = 0.03).

## Discussion

This is one of the few studies to examine SD among conflict-affected civilians in low- and middle-income countries. The findings suggest high levels of suffering, and a potentially high burden and costs of SD on individuals but potentially also health services in Georgia, which are already under resourced [41].

We observed a consistent relationship between exposure to traumatic events and SD, in line with other studies [29,35,36,50]. Specifically, serious injury, exposure to conflict situations, and experiencing the death of a family member appear to place individuals at high risk of SD. This study shows strong correlations between SD and PTSD, depression, and anxiety, again consistent with other studies [12,13,16,22,25,50], and also the links between SD and functional disability [5,12,16,17,25,41].

Female gender, economic status, education, older age, and marital status were all associated with SD, as also observed elsewhere [5,29,35,38]. In addition, being an IDP was associated with lower SD when compared with being a returnee.

How do these findings compare with the very limited literature on SD specifically among conflict-affected civilians in low- and middle-income countries? The levels of SD recorded in our study are substantially higher than recorded in the two relevant studies noted above from Bosnia [5] and Kosovo [35], which both also used the PHQ. The prevalence of SD obtained in the Bosnian study of primary care users was 16%, and it was 13% for the non-representative sample of Kosovar civilian war survivors. One explanation for the higher levels recorded in our study was that the majority of our population were still displaced from their homes and living in comparatively deprived socioeconomic circumstances. The returnees in our study had also only fairly recently returned to their home areas and continue to live under stress through impoverishment and proximity to the fragile border area. The findings in our study support this, with worse socioeconomic circumstances associated with SD. Other explanations for the higher levels of SD include the slightly higher proportion of older participants and women in our study compared to the studies in Bosnia and Kosovo (45 years versus 48, 60% female versus 65%) as levels of SD are commonly higher among older age groups and women [3]. Differences in trauma exposure may also contribute to differences in the levels reported. IDPs and returnees in Georgia also suffer from a large treatment gap for mental health services, with low levels of access and utilization [8].

These findings have implications for policy and practice. There is a need to support those providing health services in Georgia to recognize and respond appropriately to SD and to have greater understanding on the strong links between SD and physical and mental health disorders. This is particularly important given the persistently elevated levels of mental disorders among conflict-affected populations over many years [30,40]. While anyone can suffer from SD, they should be particularly vigilant with older conflict-affected people, especially women, who have been displaced for a long time and who were exposed to the most violent and traumatic events. The importance of this issue for policy lies in the evidence of increased use of health services among people with SD that has been reported from elsewhere. Thus, improved diagnosis, and subsequently more appropriate management, could improve the health, well-being, and productivity of people with SD and ensure more appropriate use of the limited resources available for health care in Georgia [9]. There is evidence, from a variety of settings, that culturally sensitive and adapted psychological approaches such as Cognitive Behavioral Therapy (CBT) can help in the management of various types of SDs and related PTSD symptoms [23,24,28,38]. It is also known that good quality social support is associated with decreased levels of psychological and SD during prolonged exile [15]. Given the resources available to support conflict-affected people in Georgia are extremely limited, it is important to ensure that what is done is culturally sensitive, effective and rigorously evaluated.

### Limitations

We were unable to differentiate somatic complaints that are psychologically related from physical complaints caused by factors such as age, somatic illness, and injury. For a diagnosis to be made with confidence, an actual medical cause needs to be eliminated, whereas the PHQ-15 focuses on somatic symptom severity regardless of cause. Without proper clinical evaluation, results from the PHQ-15 may be picking up multiple medically explained somatic complaints that may not be related to psychological distress. While the PHQ-15 has been demonstrated to be valid as screening tool for SD in a range of settings [51] and our results suggest that PHQ-15 has good reliability and validity with the study population, we did not conduct a rigorous psychometric evaluation of the main measures prior to the study. It is also important to recognize that we may not have captured somatic symptoms specific to the Georgian cultural context and how they may act as idioms of distress, and further work should take place following approaches used elsewhere [20,23,51]. Another limitation is that we did not assess use of health services by respondents at risk of SD which could have yielded valuable information on the high burden of SD for the health system. Finally, the cross-sectional design means that we are unable to observe the trends in SD or the temporal relationship between SD and mental disorders or between specific episodes such as worry episodes or panic attacks with SD [21].

## Conclusion

Our study reports high levels of SD among IDPs and returnees in Georgia, which are strongly associated with exposure to traumatic events and other mental disorders. These high SD levels indicate significant suffering. The findings also have implications for mental and physical health services in Georgia. Further research is required on SD with other conflict-affected civilian populations settings to better understand levels of SD, implications for health services, and the effectiveness of mental health interventions addressing it.

## Conflict of interest

None.

## Ethical standards

The authors assert that all procedures contributing to this work comply with the ethical standards of the relevant national and institutional committees on human experimentation and with the Helsinki Declaration of 1975, as revised in 2008.

## Competing interests

The authors have no competing interests to report.

## Figures and Tables

**Table 1:** Sample characteristics.

	All		Male		Female	
	*N*	(%)	*N*	(%)	*N*	(%)
Gender	3600	(100)	1,248	(34.67)	2,352	(65.33)

*Age (years)*						
18–39	1,274	(35.39)	434	(34.78)	839	(35.67)
40–64	1,239	(34.42)	455	(36.46)	784	(33.33)
> 65	1,087	(30.19)	358	(28.69)	729	(30.99)

*Marital status*						
Married	2,278	(63.33)	858	(68.75)	1420	(60.37)
Single	676	(18.79)	308	(24.68)	368	(15.65)
Widowed	643	(17.88)	82	(6.57)	561	(23.85)

*Education*						
Higher	723	(20.09)	236	(18.91)	487	(20.71)
Secondary	2,500	(69.48)	870	(69.71)	1630	(69.30)
> Primary	375	(10.42)	142	(11.38)	233	(9.91)

*Household economic status*						
Good	74	(2.06)	28	(2.24)	46	(1.96)
Average	1,652	(45.91)	577	(46.23)	1075	(45.71)
Bad	1,872	(52.03)	642	(51.44)	1230	(52.30)

*IDP status*						
Returnees	1,200	(33.33)	431	(34.54)	769	(32.70)
Old	1,200	(33.33)	416	(33.33)	784	(33.33)
New	1,200	(33.33)	401	(32.13)	799	(33.97)

*Mental health status*						
PTSD^a^	833	(23.48)	241	(19.31)	592	(25.17)
Depression^b^	460	(12.78)	128	(10.26)	332	(14.12)
Anxiety^c^	373	(10.75)	106	(8.49)	267	(11.35)

*Cumulative trauma exposure*						
No events	713	(19.81)	195	(15.63)	518	(22.02)
1 event	891	(24.75)	301	(24.12)	590	(25.09)
2 events	774	(21.50)	254	(20.35)	520	(22.11)
≥ 3 events	1222	(33.94)	498	(39.90)	724	(30.78)

PTSD, post-traumatic stress disorder.

a TSQ score > 5.

b PHQ-9 score ≥ 10.

c GAD-7 score ≥ 10.

**Table 2:** Levels of somatic distress, by gender (*N* = 3600).

	Total sample		Men*		Women^a^	
	*N*	(%)	*N*	(%)	*N*	(%)
At SD risk^b^	3600	(41.67)	369	(29.56)	1221	(51.91)

*SD severity:*						
Minimal^c^	1846	(51.27)	803	(64.30)	1043	(44.34)
Low^d^	1110	(30.83)	311	(24.91)	799	(33.97)
Medium^e^	523	(14.5)	110	(8.81)	413	(17.55)
High^f^	121	(3.36)	24	(1.92)	97	(4.12)

SD, somatic distress.

a All results between men and women statistically significantly different at *p* < .01 using chi^2^. Percentages are within gender group.

b PHQ-15 score > 5.

c PHQ-15 scores 0–4. ^d^PHQ-15 scores 5–9. ^e^PHQ-15 scores 10–14. ^f^PHQ-15 scores 15–30.

**Table 3:** Prevalence of the trauma exposure events in those with and without somatic distress.

Trauma exposure event	*N* (%)^a^	% with SD^b^	% without SD^b^
Lack of shelter	1609 (44.69)	47.33	42.81⁎⁎
Serious injury	594 (16.5)	23.33	11.62⁎⁎⁎
Combat situation	1651 (45.86)	55.27	39.14⁎⁎⁎
Physical abuse from family member	95 (2.64)	3.4	2.1⁎
Sexual abuse	6 (0.17)	0.27	0.1
Abduction	46 (1.28)	1.47	1.14
Torture	52 (1.44)	2.07	1⁎⁎
Murder/torture of family or friends	709 (19.69)	22.4	17.76⁎⁎⁎
Murder/torture of strangers	217 (6.03)	8.8	4.05⁎⁎⁎
Death family member during conflict	898 (24.94)	28.13	22.67⁎⁎⁎
Death family member after conflict	655 (18.19)	22.4	15.19⁎⁎⁎
Death family member non-conflict related	1011 (28.08)	32.6	24.86⁎⁎⁎

SD, somatic distress.

⁎ *p* < 0.05.

⁎⁎ *p* < .01.

⁎⁎⁎ *p* < 0.001.

a Percentage of total sample who experienced trauma exposure event.

b Prevalence of the trauma exposure events in those with (PHQ score > 5) and without somatic distress.

**Table 4:** Association of trauma exposure, mental health, and sociodemographic characteristics with being at risk of somatic distress (*N* = 3600).

	*N*	(%)	Bivariate model			Multivariate model		
			OR	[95% CI]	*p*	OR	[95% CI]	*p*
*Trauma exposure events*^a^								
Lack of shelter	1609	(44.69)	1.20	[1.05;1.37]	0.007	0.79	[0.66;0.93]	0.01
Serious injury	594	(16.5)	2.31	[1.93;2.77]	< 0.001	1.81	[1.45;2.27]	< 0.001
Combat situation	1651	(45.86)	1.92	[1.67;2.2]	< 0.001	1.66	[1.39;1.97]	< 0.001
Murder/torture of strangers	217	(6.03)	2.28	[1.72;3.03]	< 0.001	1.44	[1.00;2.07]	0.05
Death family member (non-conflict related)	898	(24.94)	1.46	[1.26;1.69]	< 0.001	1.26	[1.05;1.51]	0.02

*Cumulative trauma exposure events*								
No events	713	(19.8)	ref			ref		
1 event	891	(24.75)	1.31	[1.03;1.66]	0.03	1.31	[0.98;1.75]	0.07
2 events	774	(21.5)	1.75	[1.35;2.27]	< 0.001	1.56	[1.14;2.13]	0.01
> 3 events	1,222	(33.94)	2.44	[1.89;3.14]	< 0.001	2.02	[1.46;2.80]	< 0.001

*Mental disorders*								
PTSD^b^	833	(23.48)	5.27	[4.44;6.25]	< 0.001	2.66	[2.04;3.46]	< 0.001
Depression^c^	460	(12.78)	8.24	[5.94;11.44]	< 0.001	3.87	[2.73;5.48]	< 0.001
Anxiety^d^	373	(10.75)	5.45	[4.24;7.00]	< 0.001	1.76	[1.24;2.50]	< 0.001

*IDP status*								
Returnees	1,200	(33.33)	ref			ref		
1990s IDPs	1,200	(33.33)	0.89	[0.69;1.16]	0.38	0.60	[0.43;0.83]	< 0.001
2008 IDPs	1,200	(33.33)	0.83	[0.64;1.06]	0.13	0.72	[0.54;0.97]	0.03

*Gender*								
Male	1,248	(34.67)	ref			ref		
Female	2,352	(65.33)	2.28	[1.93;2.69]	< 0.001	2.51	[2.10;2.99]	< 0.001

*Age*								
18–39	1,274	(35.39)	ref			ref		
40–64	1,239	(34.42)	2.70	[2.27;3.20]	< 0.001	2.09	[1.70;2.57]	< 0.001
> 65	1,087	(30.19)	5.57	[4.66;6.67]	< 0.001	4.07	[3.18;5.22]	< 0.001

*Education level*								
Higher	723	(20.09)	ref			ref		
Secondary	2,500	(69.48)	1.38	[1.12;1.70]	< 0.001	1.09	[0.87;1.36]	0.44
Primary/< secondary	375	(10.42)	1.49	[1.07;2.07]	0.02	0.66	[0.46;0.95]	0.03

*Marital status*								
Married	2,278	(63.33)	ref			ref		
Single	676	(18.79)	0.52	[0.41;0.65]	< 0.001	0.68	[0.54;0.86]	< 0.001
Widowed	643	(17.88)	2.77	[2.23;3.43]	< 0.001	1.10	[0.87;1.39]	0.41

*Household economic status*								
Good/very good	74	(2.06)	ref			ref		
Average	1,652	(45.91)	1.97	[0.98;3.99]	0.06	1.58	[0.68;3.69]	0.29
Bad/very bad	1,872	(52.03)	5.03	[2.48;10.23]	0.00	2.44	[1.06;5.62]	0.04

IDP, internally displaced persons; PTSD, post-traumatic stress disorder; SD, somatic distress. Main multivariate regression model is on association of risk of SD (PHQ-15 Score > 5) with variables of gender, age, education, economic status, displacement status, mental health status, and cumulative trauma events. A separate regression model was run for exposure to individual traumatic events which excluded the cumulative trauma exposure (and adjusted for gender, age, education, economic status, displacement status, mental health status). Results shown in table for gender, age, education, economic status, displacement status, mental health status are for the main regression model and were not significantly different (*p* < 0.05) between the two models. Only statistically significant results (*p* < 0.05) included in the table.

a Reference groups are no exposure.

b TSQ score > 5.

c PHQ-9 score ≥ 10.

d GAD-7 score ≥ 10.
