# Supplementary material for: Patterns of somatic distress among conflict-affected persons in the Republic of Georgia
Source: J Psychosom Res. 2015 May;78(5):466–71. doi: 10.1016/j.jpsychores.2015.01.015 (PMC4390160; doi:10.1016/j.jpsychores.2015.01.015)
Supplement: Supplementary file 1 — Supplementary tables [file mmc1.docx]

**Title: Patterns of somatic distress among conflict-affected persons in the Republic of Georgia**

**Online Appendix 1: Item-specific results on somatic distress, by gender (N=3600)**

|  | **Men (N=1248)*** | | |  | **Women (N=2352)*** | | |
| --- | --- | --- | --- | --- | --- | --- | --- |
|  | **Bothered (%):** | | |  | **Bothered (%):** | | |
|  | Not | A little | A lot |  | Not | A little | A lot |
| Stomach Pain | 77 | 18.83 | 4.17 |  | 70.32 | 24.45 | 5.23 |
| Back Pain | 63.94 | 28.37 | 7.69 |  | 46.94 | 41.67 | 11.39 |
| Pain in Arms | 60.10 | 28.53 | 11.38 |  | 45.15 | 37.03 | 17.81 |
| Headaches | 63.94 | 31.41 | 4.65 |  | 40.77 | 50.04 | 9.18 |
| Chest Pain | 77.16 | 18.91 | 3.93 |  | 65.43 | 28.32 | 6.25 |
| Dizziness | 76.28 | 20.91 | 2.8 |  | 59.44 | 36.61 | 3.95 |
| Fainting spells | 94.87 | 4.41 | 0.72 |  | 91.92 | 6.76 | 1.32 |
| Heart | 70.67 | 25.80 | 3.53 |  | 54.89 | 39.63 | 5.48 |
| Shortness of breath | 78.45 | 25.80 | 2.96 |  | 69.73 | 26.36 | 3.91 |
| Constipation | 89.90 | 8.97 | 1.12 |  | 83.59 | 14.75 | 1.66 |
| Nausea | 88.62 | 10.34 | 1.04 |  | 81.04 | 17.60 | 1.36 |
| Tiredness | 59.78 | 32.93 | 7.29 |  | 43.37 | 46.34 | 10.29 |
| Trouble sleeping | 59.05 | 31.97 | 8.97 |  | 49.28 | 39.54 | 11.18 |
| *All results between men and women statistically significantly different at P<.001 using chi^2^ test (except fainting spells which was P<0.05). | | | | | | | |

**Online Appendix 2: Results of multivariate regression analysis on association of trauma exposures, mental health, and socio-demographic variables with medium and high somatic distress (N=3600)**

|  | **N** | **(%)** |  | ***Medium somatic distress ^1^*** | | | |  | ***High somatic distress ^2^*** | | | |
| --- | --- | --- | --- | --- | --- | --- | --- | --- | --- | --- | --- | --- |
|  |  |  |  | **OR** | **[95% CI** | | ***P*** |  | **OR** | **95% CI** | | ***P*** |
| **Trauma exposure events:^3^** |  |  |  |  |  |  |  |  |  |  |  |  |
| Serious injury | 594 | 16.5 |  | 1.99 | [1.55; | 2.54] | <0.001 |  | 2.35 | [1.52; | 3.63] | <0.001 |
| Combat Situation | 898 | 24.94 |  | 1.40 | [1.13; | 1.73] | <0.001 |  |  |  |  |  |
| Death family member (non-conflict related) | 655 | 18 |  | 1.33 | [1.03; | 1.72] | 0.03 |  | 1.78 | [1.16; | 2.73] | 0.01 |
| **Cumulative trauma exposure** |  |  |  |  |  |  |  |  |  |  |  |  |
| No events | 713 | 19.81 |  | ref |  |  |  |  | ref |  |  |  |
| 1 event | 891 | 24.75 |  | 1.59 | [1.14; | 2.21] | 0.01 |  | 1.02 | [0.48; | 2.19] | 0.95 |
| 2 events | 774 | 21.5 |  | 1.79 | [1.28; | 2.50] | <0.001 |  | 1.74 | [0.85; | 3.58] | 0.13 |
| >3 events | 1,222 | 33.94 |  | 2.46 | [1.78; | 3.39] | <0.001 |  | 3.20 | [1.62; | 6.31] | <0.001 |
| **Mental Health:** |  |  |  |  |  |  |  |  |  |  |  |  |
| PTSD ^4^ | 833 | 23.48 |  | 1.88 | [1.50; | 2.35] | <0.001 |  |  |  |  |  |
| Depression ^5^ | 460 | 12.78 |  | 4.07 | [3.09; | 5.35] | <0.001 |  | 3.16 | [1.92; | 5.19] | 0.00 |
| Anxiety ^6^ | 373 | 10.75 |  | 1.38 | [1.02; | 1.86] | 0.04 |  | 1.84 | [1.11; | 3.03] | 0.02 |
| **Displacement status:** |  |  |  |  |  |  |  |  |  |  |  |  |
| Returnees | 1,200 | 33.33 |  | ref |  |  |  |  | ref |  |  |  |
| 1990s IDPs | 1,200 | 33.33 |  | 0.41 | [0.32; | 0.54] | <0.001 |  | 0.21 | [0.13; | 0.36] | <0.001 |
| 2008 IDPs | 1,200 | 33.33 |  | 0.49 | [0.38; | 0.63] | <0.001 |  | 0.38 | [0.23; | 0.62] | <0.001 |
| **Gender:** |  |  |  |  |  |  |  |  |  |  |  |  |
| Male | 1,248 | 34.67 |  | ref |  |  |  |  | ref |  |  |  |
| Female | 2,352 | 65.33 |  | 2.48 | [1.98; | 3.11] | <0.001 |  | 2.19 | [1.36; | 3.52] | <0.001 |
| **Household economic status:** |  |  |  |  |  |  |  |  |  |  |  |  |
| Good | 74 | 2.06 |  | ref |  |  |  |  |  |  |  |  |
| Average | 1,652 | 45.91 |  | 1.94 | [0.58; | 6.48] | 0.28 |  |  |  |  |  |
| Bad | 1,872 | 52.03 |  | 3.07 | [0.92; | 10.18] | 0.07 |  |  |  |  |  |
| **Education level:** |  |  |  |  |  |  |  |  |  |  |  |  |
| Higher | 723 | 20.09 |  | ref |  |  |  |  |  |  |  |  |
| Secondary | 2,500 | 69.48 |  | 1.02 | [0.79; | 1.32] | 0.89 |  |  |  |  |  |
| Primary/<Secondary | 375 | 10.42 |  | 0.55 | [0.37; | 0.82] | <0.001 |  |  |  |  |  |
| **Age:** |  |  |  |  |  |  |  |  |  |  |  |  |
| 18-39 | 1,274 | 35.39 |  | ref |  |  |  |  | ref |  |  |  |
| 40-64 | 1,239 | 34.42 |  | 2.26 | [1.71; | 2.99] | <0.001 |  | 2.21 | [1.07; | 4.58] | 0.03 |
| >65 | 1,087 | 30.19 |  | 3.48 | [2.63; | 4.61] | <0.001 |  | 4.83 | [2.41; | 9.67] | <0.001 |
| IDP, internally displaced persons; PTSD, post traumatic stress disorder; SD, somatic distress.  Main regression model is multivariate analysis of association of medium SD (PHQ-15 Score>9) with variables of gender, age, education, economic status, displacement status, mental health status, and cumulative trauma events. A separate regression model was run for exposure to individual traumatic events which excluded the cumulative trauma exposure (and adjusted for gender, age, education, economic status, displacement status, mental health status). Results shown in table for gender, age, education, economic status, displacement status, mental health status are for the main regression model and were not significantly different (P<0.05) between the two models. The same process was the applied to models for High somatic distress (PHQ-15 Score>15)  Blank cells indicate where data not significant at P<0.05  ^1^ PHQ-15 score >9. ^2^ PHQ-15 score >15. ^3^ Reference groups are no exposure. ^4^ TSQ score >5. ^5^PHQ-9 score ≥10. ^6^ GAD-7 score ≥10. | | | | | | | | | | | | |
